# Navigating the national rollout: examining Primary Care Network link workers and the integration of local social prescribing models in Redbridge

**DOI:** 10.1017/S1463423626101443

**Published:** 2026-07-21

**Authors:** Ainul Nadhirah Hanafiah, Marcello Bertotti

**Affiliations:** Institute for Connected Communities, University of East London, UK

**Keywords:** community referral, health and wellbeing, implementation, integrated social prescribing, public health, service evaluation, social determinants of health, social prescribing

## Abstract

**Background:**

Social prescribing (SP) is increasingly recognised as a key component of primary care, implemented in at least 25 countries by 2023. In England, SP has been nationally rolled out through Primary Care Networks (PCNs), though many areas, including Redbridge in East London, had pre-existing VCSE-led models. How national and local approaches integrate in practice remains underexplored.

**Methods:**

This study used a two-step qualitative design. First, documentary analysis of service planning and evaluation documents mapped the SP referral pathway in Redbridge before and after the national rollout. Second, 16 semi-structured interviews were conducted with purposively sampled stakeholders from PCNs, RedbridgeCVS, the local authority, and the Clinical Commissioning Group, exploring service integration and delivery experiences. Data were analysed using the Framework Method, combining deductive coding against predefined themes with inductive identification of emergent categories.

**Results:**

RedbridgeCVS was widely valued for its expertise, especially in managing complex cases, training link workers, and facilitating access to local services. However, integration across PCNs was inconsistent. Participants reported variation in GP engagement, referral processes, and support structures. PCN link workers faced capacity pressures, unclear role boundaries, and limited access to clinical supervision, affecting the sustainability and coherence of service delivery.

**Conclusions:**

Integrating national and local SP models requires clearer leadership, standardized referral pathways, and consistent support for link workers. Without greater alignment across primary care and VCSE partners, the full potential of SP risks being undermined. Link workers must be recognized and supported as core members of the primary care team.

## Introduction

As of 2025, at least 25 countries had introduced social prescribing (SP) at various levels (Khan *et al.*, 2023). In England, NHS launched a national rollout in 2019, recruiting 3,000 link workers to deliver SP through GP practice-led Primary Care Networks (PCNs), each serving populations of 30,000–50,000 (NHS, 2019).

This growing interest is underpinned by evidence suggesting that SP improves health and wellbeing (Pescheny *et al*., 2019), with some support for its impact on mental health (Bickerdike *et al*., 2017) and loneliness (Reinhardt *et al*., 2021), despite methodological limitations. A qualitative review on loneliness and social isolation (Liebmann *et al*., 2022) found that SP can enhance wellbeing and social connectedness but may also cause harm when there is a mismatch between patient needs and available resources. Economic evaluations show modest reductions in healthcare usage (Polley *et al*., 2023), though some studies report increases (Elston *et al*., 2019; Jones and Lynch, 2020). A large quasi-experimental study showed cost reductions (Wildman and Wildman, 2023).

Several realist reviews have focused on how SP works rather than its outcomes, identifying key mechanisms such as social capital, trust, and belonging (Tierney *et al*., 2020). Link workers can empower individuals to manage their wellbeing, but unintended consequences – like added pressure on GPs – have been noted. Husk *et al*. (2020) found that alignment between referral expectations and delivery is critical to engagement.

These insights are especially relevant given SP’s evolution from a localized intervention to a nationally commissioned service. Before the NHS rollout, many areas developed bespoke models to meet specific needs (e.g. loneliness, mild mental health conditions, or diabetes), often supported by Clinical Commissioning Groups and local authorities. Redbridge (East London) is one such case, where a VCSE-led SP model was in place before PCN link workers were introduced. It is important to assess whether national implementation has altered these local models and how link workers’ roles have evolved. To date, little research has explored how existing SP models have integrated with the national PCN approach, nor has there been much documentation of link worker experiences within this transition.

Given the diversity of definitions of SP (Muhl *et al*., 2023), this study adopts the following: SP refers to a healthcare pathway enabling GPs, and increasingly other professionals, to refer patients with psychosocial needs to non-clinical, community-based support. These referrals are typically facilitated by a link worker who provides one-to-one support, often in the form of a flexible, coaching-style intervention. Clients are usually connected to services delivered by Voluntary, Community and Social Enterprise (VCSE) organizations offering practical and emotional assistance.

To frame the analysis, this study draws on the concept of networked ecosystems, which foregrounds value co-creation across multiple actors operating at different levels of a system (Gallan *et al*., 2019; Farina *et al*., 2024). In the context of SP, this lens draws attention to the relational and organizational dynamics through which primary care, local government, and the VCSE sector collectively generate value for communities. Rather than treating integration as a technical challenge of aligning pathways, the networked ecosystem framing highlights the knowledge flows, relational trust, and shared capacities that either support or undermine effective service delivery. This study examines whether and how such an ecosystem has been cultivated in Redbridge, and what conditions appear to enable or constrain it.

This study explores how the national rollout of SP has functioned in the context of Redbridge’s pre-existing VCSE-led model, identifying the opportunities and challenges of integrating the two approaches. It focuses particularly on the experiences of PCN link workers and aims to inform both local and national policy, while contributing to wider conceptual and policy debates on the future development of SP.

### Redbridge social prescribing

In late 2017, the London Borough of Redbridge and the local Clinical Commissioning Group began funding SP through RedbridgeCVS, a key organization within the local VCSE sector. GPs typically referred individuals experiencing social isolation, loneliness, mental health issues, or Type 2 diabetes. SP advisors provided flexible, holistic support – linking individuals to VCSE activities or statutory services, facilitating referrals, and accompanying clients to sessions when needed.

An independent evaluation by Bertotti *et al*. (2020) (*N* = 182) reported significant improvements in mental wellbeing and quality of life, although reductions in loneliness were limited. Despite the availability of training and clinical supervision, challenges included high referral volumes and increasingly complex cases. During the COVID-19 pandemic, services continued remotely following the physical closure of RedbridgeCVS in early 2020.

The 2020 national rollout introduced a new referral pathway. GPs now refer patients to PCN link workers, who assess needs and either connect clients directly to services or refer them to RedbridgeCVS for further support. Since 2017, RedbridgeCVS has provided personalized support, particularly for socially isolated individuals, carers, and vulnerable groups, often including home visits and activity accompaniment. However, the introduction of PCN link workers in 2020–2021 has brought both opportunities and tensions, highlighting the need to clarify RedbridgeCVS’s role in the evolving system.

## Methods

### Design

This study utilized a two-step qualitative design to address the aims identified above: (i) investigate how service integration occurred and associated opportunities and challenges; (ii) examine the experience of PCN link workers. Qualitative research was seen as an important way to investigate the meanings and realities of different stakeholders involved in intervention delivery.

Firstly, a review of Redbridge SP documents was undertaken to identify plans for the development of the service across the borough. The documents were selected because they described the intended structure, referral routes, and role configuration of the local SP model. Documents reviewed included: RedbridgeCVS and Redbridge Council documents related to the development and planning of SP in the borough; the integrated SP proposal produced by RedbridgeCVS, which set out the intended design for integrating the VCSE-led model with incoming PCN link workers; RedbridgeCVS quarterly reports documenting service activity, referral patterns, and implementation progress; and NHS guidance on the PCN link worker model and competency framework (NHS, 2019, 2022).

**Figure 1.**
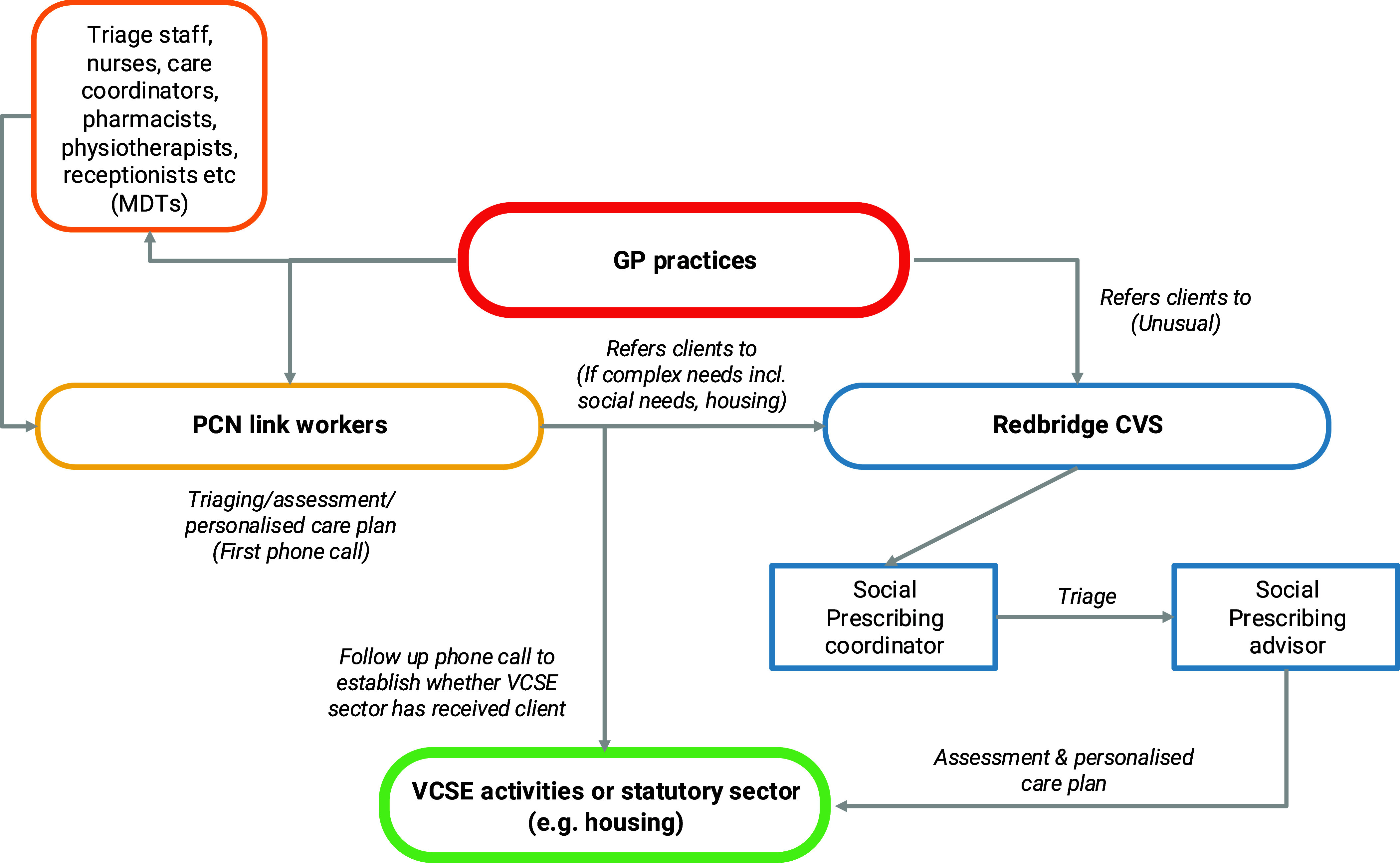
Figure 1 long description. Mapping of social prescribing pathways in Redbridge.

Data were extracted systematically from each document, with a focus on service design features, stakeholder roles and responsibilities, referral processes, and changes introduced by the national rollout. Extracted information was organized using a structured summary template to facilitate cross-document comparison. Relevant information was then used to develop an initial pathway map, which was subsequently examined and refined against interview data. The pathway map depicts the client’s journey from initial GP contact through to referral to RedbridgeCVS and onward connection to VCSE services (see Figure 1). Service users were not involved in the mapping process, as the study’s focus was on organizational integration and professional roles rather than the client experience of the pathway. Subsequently, semi-structured interviews were conducted online via Teams or telephone between May and August 2022 to explore more in-depth the experiences of those involved in the strategy or delivery of the service in the borough.

### Sample and data collection

Various stakeholders were included such as relevant SP PCN members, i.e. link workers, managers, clinical director, as well as local council member, NHS CCG representative, RedbridgeCVS SP advisors (SP advisors) and other stakeholders involved in the strategy or delivery of SP in the borough and surrounding areas. Participants were recruited using a purposive sampling strategy, designed to capture a range of perspectives across the key stakeholder groups involved in SP strategy and delivery in Redbridge. The primary inclusion criterion was direct involvement in the design, commissioning, coordination, or delivery of SP in Redbridge at any point during or after the national rollout.

Participants were identified through organizational contacts within RedbridgeCVS and the PCN network, with sampling continuing until thematic saturation was reached – defined as the point at which successive interviews yielded no substantively new themes relevant to the study’s core questions. The final sample of 16 participants spanned four stakeholder groups: PCN staff (link workers, managers, and a clinical director), RedbridgeCVS staff (service lead, coordinator, and advisors), a Clinical Commissioning Group representative, and a local authority representative. Participants referred to their local Clinical Commissioning Group, which formally became an Integrated Care Board in July 2022 during the fieldwork period; CCG is used throughout to reflect the nomenclature used by participants at the time. General practitioners, practice nurses, and allied health professionals were not included in the sample. This reflected the study’s focus on service integration between the PCN link worker tier and RedbridgeCVS; GP perspectives on referral practices were captured indirectly through the accounts of link workers, managers, and clinical leads. The absence of direct GP and allied health professional perspectives is acknowledged as a limitation (see Strengths and Limitations).

Interview guides were prepared, with the guide for PCN link workers being structured specifically around the competencies framework developed by the NHS (NHS, 2022) to account for the different aspects of link workers’ roles. Guides for other stakeholder groups were adapted to reflect their specific organizational positions, with a shared focus on service integration, referral processes, and perspectives on future development. We did not include interviews with service users as we focused on issues of service integration, particularly how PCN link workers and RedbridgeCVS advisers worked together and the general working conditions of link workers rather than focussing on the experience of service users.

### Data management and analysis

All interviews were audio recorded, and transcribed verbatim by a professional transcriber. Transcripts were uploaded into NVivo qualitative software programme, which facilitated team-based coding and subsequent data analysis.

We adopted the Framework Method analysis with the interview data as this allows exploration of diversities within a given population by providing detailed and extensive in-depth information (Ritchie *et al*., 2013). The Framework Method was selected because it is particularly well-suited to studies with predefined topics of interest – in this case, service integration and link worker experience – whilst also accommodating the exploration of diversity across multiple stakeholder groups.

Analysis followed the five stages outlined by Ritchie *et al.* (2013): familiarization with the data through reading and re-reading transcripts; identification of an initial thematic framework; systematic indexing of the data against this framework; charting of data into a matrix format within NVivo to facilitate cross-case comparison; and mapping and interpretation to identify patterns and conceptual explanations. The analysis combined deductive coding, informed by the study aims and interview guide, with inductive coding to capture themes emerging from the data. The framework was developed through familiarization, coding, charting, and interpretation, with coding discrepancies discussed and resolved within the research team.

An experienced researcher coded the interviews which were then checked, refined, and expanded by a second researcher. The team also reviewed a random selection of transcripts to ensure consistent application of theme categorizations.

### Ethical considerations

Ethical clearance from the University of East London (ETH2122-0167) was sought prior to the interviews. All respondents provided written consent before the interview commenced, and their right to decline participation at any point during the interview was informed at the start of their session.

## Results

A total of 16 in-depth qualitative interviews were conducted with a wide range of stakeholders including PCN staff at different levels, e.g. Clinical Director, managers, PCN link workers; RedbridgeCVS at different levels, e.g. Lead, coordinator and advisors; and other stakeholders from the local Clinical Commissioning Group and local authority. This was thought to provide greater depth and the opportunity to validate views offered by people at different organizational levels.

We conducted the interviews across four broad analytical themes, to explore issues of integration and the experience of PCN and RedbridgeCVS members:

1. The structure of the SP pathway.
2. Integration of SP service.
3. Suggestions for future improvement of an integrated SP model.

Firstly, we present the mapping of pathway of SP in Redbridge, followed by the experience in providing the service which includes the coverage of service and expectations towards link workers and SP advisors. In the second part of the results, we focus on the strengths and weaknesses of the integrated SP in Redbridge. Finally, we include respondents’ views on the future developments of an integrated SP model.

### The structure of the social prescribing pathway in Redbridge

This section outlines the client’s journey from their initial GP visit to referral to PCN link workers, then to a SP advisor, and finally to delivery organizations, including VCSE and statutory sectors like housing – see Figure 1. Since the focus is on SP integration in Redbridge, the onward referral to the VCSE sector, common to all SP models, is not covered. Interviews primarily focused on the perspectives of stakeholders from PCN and RedbridgeCVS, who were actively involved in the service provision.

#### The first stage of the pathway: referral from GP practice to PCN link workers

Before the 2020 national rollout, GPs in Redbridge referred patients directly to RedbridgeCVS SP advisors, who then connected them to relevant VCSE activities or statutory services. The introduction of PCN link workers created an additional tier within the pathway: GPs now refer to link workers in the first instance, who assess clients’ needs and either address them directly or refer more complex cases to RedbridgeCVS.

This structural change effectively repositioned link workers as a gatekeeping and triage function between primary care and the CVS, adding a stage to the referral process and altering the nature of the relationship between GP practices and the VCSE sector. Figure 1 illustrates both the pre-2020 model and the current integrated pathway, demonstrating how the introduction of the PCN link worker role has reshaped the flow of referrals across the system.

A range of healthcare professionals – including GPs, nurses, physiotherapists, pharmacists, and even receptionists – referred patients to SP. Most referrals came directly from GPs or via Multi-Disciplinary Team (MDT) meetings involving PCN link workers. Pharmacists also contributed referrals through Structured Medication Reviews, identifying patients with complex social and medication needs.

Link workers consistently highlighted the importance of building strong relationships with GP practices, though they reported considerable variation in referral volume and type (e.g. welfare-related). Despite promotional efforts, many expressed frustrations over inconsistent engagement. Most spent significant time cultivating and maintaining these relationships, which required repeated outreach and were sometimes hampered by limited GP communication.

Strategies included brief presentations at weekly practice meetings, as well as posters, leaflets, and regular updates on patient outcomes. Some link workers also involved practice managers and administrative staff in referral pathways. RedbridgeCVS had previously employed similar engagement methods, and since integration, its coordinators have shifted focus to working directly with link workers and care coordinators across PCNs rather than only with GPs.

It was noted that a key barrier to embedding SP was the lack of understanding about the service and the link worker role. Given the interconnected nature of the service, maintaining relationships was seen as essential to success. One link worker described the repetitive but necessary process of reinforcing their presence:

*‘So, it was kind of like reminding them that we’re here again and again and I did feel like it was a bit repetitive but I think they were just trying to grasp the concept of social prescribing and er eventually they did and then they started referring and started referring more and more because they realized, ‘Okay this is what social prescribing is, it’s actually helping’. (RSP08; PCN link worker)*

Link workers agreed that sustained engagement with GP practices was crucial but often challenging, as GPs were overwhelmed with competing demands and tended to deprioritise SP. Despite ongoing efforts, engagement was inconsistent. Link workers also participated in MDTs to identify patients’ needs and support appropriate referrals, though referral rates varied widely across PCNs.

#### The second stage of the pathway: conditional referral from PCN link workers to RedbridgeCVS

PCN link workers typically received the majority of GP referrals and passed on more complex cases to RedbridgeCVS for statutory support (e.g. housing, social care). At RedbridgeCVS, the SP coordinator triaged incoming cases and assigned them to an advisor based on their needs. As one advisor explained:

*‘The pathway is very similar [to previous setup], it’s just that most of our referrals now tend to come from the [PCN] link workers. And the GPs will pass to them, and they will almost act as a sort of triage I would imagine, even though it’s not called that. And anything that’s going to require a lot of time or things that they don’t deal with then they pass them to us’. (RSP31; RedbridgeCVS)*

CVS respondents noted variation in referral volumes across PCNs, with more than half of referrals coming from a single network. The complexity of referrals also increased, particularly those involving housing needs or vulnerable groups such as refugees and homeless individuals. As a result, CVS began to accept fewer referrals, given the intensive time required per case.

Referral reasons varied. Some link workers referred based on session length, type of activity, or when clients required support beyond borough boundaries. CVS was perceived to have greater capacity and local expertise, and some link workers referred clients requiring longer-term intervention to CVS, which was seen as better equipped to meet those needs.

Participants offered several explanations for the variation in referral volumes across PCNs. The most frequently cited factor was the degree of GP engagement: link workers based in PCNs where GPs were actively involved in SP – attending MDT meetings, making consistent referrals, and communicating outcomes – reported higher and more stable referral flows. Structural differences across PCNs also played a role, with some having more systematic processes for identifying eligible patients through Structured Medication Reviews or routine consultations, whilst others relied primarily on ad hoc referrals from individual clinicians.

Link workers also noted that they managed a considerable volume of cases without making a formal referral to RedbridgeCVS – typically where a client’s needs could be addressed through a single signposting contact, a brief intervention, or direct connection to a community activity. These lighter-touch encounters did not always generate a formal referral record, contributing to the difficulty of establishing an accurate picture of total caseload and activity across the system.

### Integration of social prescribing service

Following the overview of the referral pathway and link worker experiences under the national SP model, this section explores the integration between RedbridgeCVS and PCN link workers, drawing on stakeholder perspectives of how implementation has unfolded across the borough.

#### Strengths of current model

*The value added of RedbridgeCVS*. Most PCNs recognized the important contribution of RedbridgeCVS, particularly in handling complex cases. Beyond addressing vulnerability, CVS was seen as crucial in enabling access to key services – especially council-linked support – due to its long-established local relationships.

*‘The CVS has really taught us everything we know, because the PCN didn’t have any knowledge. […] They’ve trialled it well before us’. (RSP02; PCN link worker)*

CVS staff and PCN managers confirmed this value, noting their ability to manage complex referrals, link clients to foodbanks and welfare services, and provide in-depth, ongoing support – often beyond the capacity of individual link workers.

*‘Anything complex that requires a bit more hand holding than what we can initially provide, we have that service through RedbridgeCVS’. (RSP23; PCN manager)*

The breadth of CVS support was especially helpful when clients lived outside borough boundaries or required sustained input. In addition to handling referrals, RedbridgeCVS also trained new link workers and acted as a single point of access to other VCSE organizations. This role helped reduce duplication and supported smaller VCSE providers who lacked the capacity to handle multiple direct enquiries.

*Fostering collaboration and knowledge sharing in a networked ecosystem.* A key strength of the Redbridge model was its knowledge-sharing ecosystem. PCN link workers helped bridge primary care with wider services, improving collaboration between professionals such as mental health nurses, pharmacists, Citizen’s Advice, and drug and alcohol teams. Regular PCN meetings facilitated integration into MDTs, allowing link workers to contribute to joined-up care planning. In some PCNs, a blended approach enabled healthcare staff and link workers to cross-refer clients and address both clinical and social needs.

RedbridgeCVS played a pivotal role in this ecosystem by mentoring link workers, supporting complex cases, and sharing knowledge about local services. Stakeholders described it as a hub that built capacity across the system and supported smoother collaboration. SP was also seen as adding capacity to general practice by tackling non-medical issues that often complicate clinical care.

*‘The CVS service has been a great service and has shown real collaboration across the Borough… it definitely brought social prescribing to the forefront of the PCNs’ minds’. (RSP27; CCG)*

*‘Your social prescriber manages to, not just sort out their housing needs, but their benefits, their appointments for the hospital, their transport systems… the amount of time that it frees up is just amazing’. (RSP16; Clinical Director)*

#### Challenges of current model

Despite its strengths, the current SP model faces several notable challenges.

*Evolving roles and challenges in the partnership with RedbridgeCVS.* Most PCN link workers acknowledged RedbridgeCVS’s extensive experience in SP. However, one PCN manager believed that, over time, link workers would gain sufficient expertise to manage complex cases independently, potentially reducing the need for CVS involvement. Initially, RedbridgeCVS – funded by HealthBridge – supported PCNs through training and monthly meetings, which link workers found valuable, though many later struggled to attend due to increasing workload. Some link workers, managers, and a clinical director raised concerns about delays in referrals and rejections from CVS based on client suitability. Others noted that CVS’s strict inclusion criteria often led to rejected referrals. Some felt CVS was not always regarded as an equal partner by primary care, with limited understanding among PCN staff of the VCSE sector’s role and its value in supporting residents with complex needs.

Underlying some of these tensions were structural differences in how PCN link workers and RedbridgeCVS were funded and held accountable. PCN link workers, funded through the NHS Additional Roles Reimbursement Scheme, operated within NHS performance frameworks with defined targets and throughput expectations. RedbridgeCVS, funded through a separate local authority and CCG arrangement, operated under a different set of expectations oriented towards holistic, longer-term support with less rigid activity metrics. These divergent accountability structures shaped how each part of the system defined a successful interaction, creating implicit tensions around case complexity, throughput, and the appropriate scope of work for each tier.

As link workers gained confidence and experience, questions arose about where the boundaries of their role ended and those of CVS advisors began – particularly in relation to housing casework, benefits navigation, and longer-term befriending support. This ambiguity was not consistently resolved through formal guidance, and in some cases contributed to link workers bypassing RedbridgeCVS in favour of direct referrals to external services, further blurring the boundaries between health, social care, and VCSE responsibilities.

One PCN manager suggested a more integrated approach – leveraging CVS’s expertise through a consultancy model rather than relying solely on formal referrals:

*‘As far as I’m concerned what we should have been doing is looking at the CVS model and saying, ‘Right you’ve got some key experienced people […] can we buy some of that key experiences person time to actually supervise all of our people?’’ (RSP21; PCN manager)*

These reflections highlight a shared interest in strengthening collaboration between PCNs and RedbridgeCVS, with the goal of expanding and improving SP services across the borough.

*Capacity constraints and lack of clarity about roles.* PCN link workers often covered large areas – managing up to eight GP surgeries and serving populations as high as 82,000 – which translated to caseloads of up to 1,000 clients. This significantly stretched their capacity, resulting in reduced personalization of support and increased risk of burnout.

*‘I cover seven surgeries […] and I’m the only social prescriber’. (RSP01; PCN link worker)*

In some cases, link workers returned from leave only to be immediately assigned overwhelming workloads, such as supporting 50 bereaved patients. NHS guidelines suggest a manageable caseload is around 200–250 per year yet link workers in some networks were expected to manage up to 840. A PCN manager highlighted how such pressures compromised the quality of support, especially when expectations limited link workers to brief interactions:

*‘You cannot safe space an individual and form a relationship in X amount of minutes. […] You’ll get what I call the revolving door’. (RSP21; PCN manager)*

A further challenge was the inconsistency in role expectations across PCNs. Some link workers received pre-triaged referrals, while others were assigned broad, impersonal tasks, such as cold-calling hundreds of patients based on BMI listings.

*‘We just get print offs from the GPs […] and they just say, ‘Work your way through them’.(RSP02; PCN link worker)*

This lack of role clarity often left link workers operating beyond their original remit, with limited boundaries and support, contributing to role strain and dissatisfaction.

*Supervision and support for service providers.* Respondents consistently highlighted the lack of clinical supervision, stressing the urgent need for structured support in delivering SP services. Despite working with vulnerable individuals and managing complex cases, link workers reported little to no supervision, alongside confusion about who should provide it, how often, and in what format.

*‘I had to fight for it, it took me over a year […] I went off sick with stress […] they said we could have another three months but that’s finished, so no, no longer’. (RSP02; PCN link worker)*

*Clarity, leadership and system integration of SP.* Two PCN managers observed a limited understanding of SP among some clinical staff, particularly around the role and value of link workers. Most respondents described the service as fragmented, with unclear leadership and a lack of recognition – for example, of the role played by the RedbridgeCVS coordinator. Other challenges included inconsistent data collection across PCNs and CVS, limiting shared learning and evaluation. Broader systemic issues such as social determinants of health and persistent inequalities were also seen as barriers to effective service development. One clinical director summarized the growing expectations placed on link workers:

*‘There’s a real risk that this, you know, is that you know, ‘the social prescribers they can deal with it all.’ And unfortunately, they can’t […] that’s what needs to be fed back to the politicians and the ICSs and the teaching systems’. (RSP16; Clinical Director)*

### Suggestions for future improvement of an integrated social prescribing

Respondents were invited to share their recommendations for strengthening SP across the borough. This section highlights four key themes that emerged, focused on enhancing the quality, coherence, and sustainability of existing care and support.

#### Need for recognized leadership and strategic direction

Respondents placed particular emphasis on the need for recognized leadership across PCN and RedbridgeCVS SP teams. The current model in Redbridge was widely seen as lacking direction and cohesion due to the absence of a dedicated figure to provide oversight and coordination on behalf of the PCNs. In addition to leadership, respondents stressed the importance of mapping service gaps across PCNs and clarifying the role of RedbridgeCVS in addressing these. These points reflect a shared desire for greater coherence, communication, and accountability across the local SP system.

#### Establish standardized pathways and impact assessment for effective social prescribing

Respondents strongly emphasized the need for clearer leadership, a standardized referral pathway, a shared database of available services, and robust evaluation of cost-effectiveness and health outcomes. These measures were seen as essential for ensuring consistent, high-quality experiences for both service users and providers.

*‘I would want a consistency of protocol and process […] because it saves a hell of a lot of misunderstanding space and then you can work in a much more structured framework’. (RSP21; PCN manager)*

*‘I’d like to see a clear and consistent pathway so all residents in Redbridge get the same sort of service. […] so patients are clear about how they access the service’. (RSP27; CCG)*

From a commissioning perspective, demonstrating value for money was also a key concern. Respondents highlighted the importance of gathering evidence that the service delivers measurable impact.

#### Integrating social prescribing into integrated care strategies

The future of SP within the emerging Integrated Care System (ICS) structures remains uncertain. One respondent noted the likelihood of increased centralization but emphasized the importance of maintaining locally tailored delivery that reflects the borough’s diverse sociodemographic and ethnic profile. Greater integration with ICSs could also broaden referral routes – from sectors such as physiotherapy – which was viewed positively.

*‘Social prescribing is much needed. […] more people are able to get help of different types, that they might not have got otherwise, or they might not have known where to turn to’. (RSP29; RedbridgeCVS)*

Respondents also highlighted that workforce planning at the ICS level could improve the identification of system-wide needs and provide more targeted training, including leadership development and SP-specific support.

## Discussion

### Summary and comparison with existing literature

In examining the collaborative relationship between RedbridgeCVS and PCN link workers, respondents consistently recognized RedbridgeCVS for its expertise in SP, capacity to support complex cases, delivery of additional sessions, training of new link workers, and facilitation of collaboration across a networked ecosystem. This included linkages with healthcare professionals, local authority services, and VCSE organizations. The concept of a *networked ecosystem* offers a useful policy and conceptual frame for service integration – one that promotes value co-creation across different levels of the system (Gallan *et al.*, 2019; Farina *et al.*, 2024), with Redbridge offering a practical illustration of such an approach.

Redbridge represents a distinctive case within the national rollout for two reasons. First, it is among the few areas to have had a mature, funded VCSE-led SP model in place before the introduction of PCN link workers, meaning the national rollout did not create a service from scratch but rather introduced a new professional tier into an established system. This created an integration challenge of a specific kind: negotiating roles, referral flows, and accountability between two existing service configurations, rather than simply embedding a new role within primary care. Second, the long-standing presence of RedbridgeCVS provided a platform for knowledge transfer, training, and cross-sector collaboration that is not universally available, making the positive features of the model as much a product of organizational history as of the national policy framework. These features mean the Redbridge experience offers both instructive lessons and important caveats for other settings.

The concept of a *knowledge-sharing ecosystem* – developed inductively from these findings and building upon the networked ecosystems framework introduced in this paper – describes a specific mechanism in which a mature VCSE organization actively builds capacity, circulates expertise, and enables coordination across a heterogeneous network of primary care providers. This is, to our knowledge, an original conceptual contribution of this study, distinct from but related to the broader notion of learning environments, which typically describes structured pedagogical settings. The knowledge-sharing ecosystem operates informally and relationally, through mentoring, joint working, and the brokering of knowledge about local services, and may offer a productive lens for understanding VCSE – primary care integration in other settings.

However, RedbridgeCVS also faced criticism for inefficiencies in referrals, client rejections, and its unclear role within primary care. While initial support and training were valued, some link workers increasingly operated independently and, in some cases, bypassed CVS for direct referrals to external services. Several respondents noted a lack of parity between RedbridgeCVS and primary care teams, reflecting broader gaps in leadership and strategic guidance needed to drive integration.

A key finding from this study is the highly variable implementation of SP across PCNs. Despite the national rollout providing guidance and resources, delivery models within the same local authority differed considerably. GP buy-in varied, and support offered ranged from brief, 15-minute consultations to structured programmes lasting several sessions over three months.

Link workers faced pressure to deliver ‘light-touch’ interventions due to performance targets and funding constraints, often at the expense of in-depth, relationship-based support. Many reported excessive caseloads, lack of clarity around role expectations, inadequate supervision, and unsatisfactory working conditions – issues echoed in other studies (e.g. NALW, 2020; Tierney *et al*., 2024; Mulholland *et al*., 2025).

These constraints contributed to high staff turnover in some PCNs. As Tierney *et al.* (2024) note, sustained investment is needed to embed the link worker role within primary care. Without this, link workers risk being viewed as peripheral rather than integral members of the care team, limiting their effectiveness and job satisfaction.

Capacity issues have also led to limited follow-up with clients, creating a ‘revolving door’ effect. In some PCNs, this was compounded by the prioritization of other GP practice demands. Tierney *et al.* (2020) highlight that link workers are instrumental in building social capital – fostering trust, belonging, and motivation – yet these benefits are undermined when capacity is constrained.

Underlying many of these challenges is a lack of shared understanding of SP and the link worker role. Evidence suggests that successful engagement is more likely when referrals align with patient expectations and are supported by trusted professionals (Husk *et al.*, 2020; Liebmann *et al.*, 2022). Therefore, establishing clear referral pathways, ensuring adequate access to VCSE and statutory services, and providing structured leadership should be priorities for future improvements.

This case study of Redbridge demonstrates the need for a more coherent and coordinated approach to supporting link workers across PCNs. While national organizations focus on evidencing health outcomes, cost-effectiveness, and funding for VCSE delivery, our findings underscore a more immediate need: developing an integrated, consistent model of care that places link workers at the heart of SP.

### Strengths and limitations

This study offers several notable strengths. It is the first to examine the integration of SP within the context of the national rollout, generating findings of clear relevance to policy and practice. Methodologically, the two-step design combining documentary analysis with qualitative interviews enabled triangulation of formal service design with reported practice. The inclusion of diverse stakeholders across organizational levels – PCN link workers, managers, clinical leads, CCG representatives, and local authority staff – provided the analytical depth needed to compare perspectives and validate findings across positions within the system.

However, the absence of interviews with SP users and VCSE representatives is a potential limitation. Given the study’s focus on implementation rather than outcomes, this was considered an appropriate trade-off. Additionally, the study’s single-site, time-bound case study design may limit the generalizability of findings. The value of single-site qualitative case studies in implementation research lies not in statistical representativeness but in the depth with which they illuminate implementation processes and mechanisms – the how and why of integration, rather than simply whether it works. Drawing on NICE guidance on the applicability of public health evidence, several dimensions of transferability can be considered. In terms of population, Redbridge serves a diverse, urban, multi-ethnic population with significant socioeconomic deprivation, a profile shared by many other urban primary care settings across England and internationally which also either offer or may offer SP in the future. In terms of setting, the structural challenge examined here – integrating a nationally mandated primary care role with an established VCSE network – is not unique to Redbridge; it is the defining implementation problem of the national SP rollout and directly relevant to the majority of PCNs in England.

In terms of intervention, the PCN link worker model is nationally consistent, and the referral dynamics, role boundary ambiguities, and supervision deficits identified here have been independently reported in other settings (Frostick and Bertotti, 2019; NALW, 2020; Tierney *et al.*, 2024; Westlake *et al*., 2024; Mulholland *et al.*, 2025), lending credibility to the wider applicability of these findings. In terms of theory, the knowledge-sharing ecosystem framework developed here is not bound to the Redbridge context and may be applied to examine VCSE–primary care integration more broadly. Future research across multiple sites and time points is nevertheless needed to strengthen external validity and assess whether the conditions observed in Redbridge can be replicated or approximated elsewhere. The absence of direct GP and allied health professional perspectives is also acknowledged as a limitation: their inclusion in future studies would provide a more complete account of integration barriers and cross-professional dynamics, particularly around referral variation and MDT engagement.

### Practical implications and recommendations

Guided by data from this study and that of Tierney *et al.* (2024) on what contributes to successful role of PCN link workers in primary care, recommendations include:

1. **Standard Operating Procedure (SOP):** Develop a jointly created SOP for all stakeholders, including criteria to assess client complexity and defined pathway for support on complex cases.
2. **Improved information sharing:** Establish a comprehensive database of support services across, e.g. PCNs, VCSE, CCG, local authority.
3. **Leadership in SP:** Appoint structured leadership in the integrated SP model, leveraging on experience and expertise. Address concerns by finding a middle ground, potentially involving a clinical representative to enhance collaboration.
4. **Data collection and analysis:** Standardize data collection systems to evaluate health and social outcomes, leveraging ongoing initiatives. In the short term, analyse data from selected PCNs to assess effectiveness and guide future investments.
5. **Clinical supervision for PCN link workers:** Pool resources, e.g. from PCNs, the CCG to employ a clinical supervisor for psychological support and safeguarding advice for PCN link workers.

## Conclusions

An ageing population, the long-term effects of the pandemic, and economic pressures such as the cost-of-living crisis have contributed to a rise in complex cases that demand a coordinated response across primary care, local authorities, and the VCSE sector. The integration of SP, grounded in the co-creation of value across multiple levels of the health and care ecosystem, represents a promising conceptual and policy direction. The collaboration between MDTs, PCN link workers, and RedbridgeCVS illustrates how such an ecosystem can align health and social support to more effectively address health inequalities.

However, this paper argues that a key limitation of the national rollout is the fragmented implementation of SP by PCNs. Inconsistent support structures, growing performance targets, and poor working conditions – including lack of clinical supervision – have hindered the capacity of some link workers to deliver high-quality care. The study identifies practical responses to these challenges, with implications for strengthening SP in England and beyond. Ultimately, SP should be seen not only as a service intervention but as a transformative approach to fostering collaboration and integration across the healthcare system.

## Data Availability

The datasets used during the current study are available from the corresponding author on reasonable request.

## Figure 1. Long description

A flowchart illustrating the process of social prescribing in England. The flowchart starts with GP practices, which refer clients to PCN link workers or Redbridge CVS if complex needs including social needs and housing are involved. PCN link workers handle triaging, assessment, and personalized care plans during the first phone call. They follow up to establish whether the VCSE sector has received the client. GP practices can also refer clients to Redbridge CVS, which involves a social prescribing coordinator and a social prescribing advisor for triage and assessment and personalized care plan.

Navigate back to Figure 1..
